# Neuroendocrine characterization into schizophrenia: norepinephrine and melatonin as promising biomarkers

**DOI:** 10.3389/fendo.2025.1551172

**Published:** 2025-05-01

**Authors:** Junwei Shen, Xin Li, Yinghua Zhong, Jiechun Zhang, Hongyun Qin, Fazhan Chen, Xudong Zhao

**Affiliations:** ^1^ Clinical Research Center for Mental Disorders, Shanghai Pudong New Area Mental Health Center, School of Medicine, Tongji University, Shanghai, China; ^2^ Department of Physical Examination Center, Shanghai East Hospital, School of Medicine, Tongji University, Shanghai, China

**Keywords:** schizophrenia, neuroendocrine marker, BDNF, norepinephrine, melatonin, metabolism

## Abstract

**Background:**

Although brain-derived neurotrophic factor (BDNF)has garnered extensive attention as a neuroendocrine marker in schizophrenia (SZ), its clinical utility remains limite due to inconsistent findings.

**Methods:**

To address this gap, serum samples were collected from 24 female patients with SZ and 25 healthy controls. The metabolic profiling was performed using gas chromatography-mass spectrometry (GC-MS) and liquid chromatography-mass spectrometry (LC-MS) to capture abroad range of metabolites.

**Results:**

Our results revealed that BDNF is not a robust discriminatory biomarker. Marked differences in metabolic profiles were identified between patients with SZ and healthy individuals. The GC-MS analysis revealed significant differences in 79 metabolites; while the LC-MS analysis identified 419 significantly differential metabolites. Functional analysis reveals that these differential metabolites predominantly contribute to metabolic and neuro-related processes. Our findings demonstrate that norepinephrine and melatonin, two additional neuroendocrine compounds, are significantly elevated in patients with SZ compared to healthy controls. Notably, their higher areas under the curve (AUC) values compared to BDNF highlight their potential as more reliable biomarkers for SZ.

**Conclusion:**

This study offers valuable insights into the altered metabolic patterns of female patients with SZ and establishes melatonin and norepinephrine as promising neuroendocrine biomarkers, underscoring their diagnostic value and role in the neuroendocrine regulation of mental disorders.

## Introduction

Schizophrenia (SZ) is a severe mental disorder characterized by delusions, hallucinations, disorganized speech, and changes in motivation and cognition, as well as emotional symptoms (1–3). As of 2022, the World Health Organization (WHO) reports that approximately 24 million (0.32%) individuals globally have been diagnosed with SZ (https://www.who.int/news-room/fact-sheets/detail/schizophrenia). Notably, treatment outcomes usually fall greatly short of expectations. A systematic review indicates that only 13.5% of patients with SZ meet the clinical and social rehabilitation criteria, contributing to SZ being recognized as the 12th leading cause of disability globally (4, 5). Alongside its early onset and chronic progression, SZ imposes a heavy burden on economies and societies, causing an estimated annual economic loss of over $215.1 billion in the United States alone (6).

The current diagnosis of SZ is primarily symptom-based, as per the World Health Organization’s eleventh version of the International Classification of Diseases (ICD-11) and the fifth edition of the Diagnostic and Statistical Manual of Mental Disorders (DSM-5) issued by the American Psychiatric Association (7–9). Both sets of criteria involve the presence of two or more characteristic symptoms coupled with social or occupational dysfunction. However, these diagnostic criteria are limited because they depend heavily on subjective assessments and symptom descriptions, lacking the support of specific biomarkers or imaging studies (10). Additionally, differentiating SZ from other mental disorders with similar symptomatology can be challenging, such as bipolar disorder, which may present with SZ-like episodes in about 50% of patients (11, 12).

Extended research into alternative diagnostic methods for SZ has been conducted, fostering the potential use of biomarkers (13–16). Among them, brain-derived neurotrophic factor (BDNF), a neuroendocrine marker, is considered one of the most promising candidates (17, 18). BDNF is a protein found in the central nervous system, essential for the growth, development, maintenance, and plasticity of neurons (19). By binding to its high-affinity receptor TrkB, BDNF promotes neuronal survival, synapse formation, and signal transduction (20). Some meta‐analyses and clinical studies have reported that patients with SZ tend to have lower serum BDNF levels than healthy controls (21, 22). However, several studies found no significant differences or even increased serum BDNF levels in patients with SZ, suggesting that BDNF’s alterations may depend on factors such as sex, illness stage, and sample characteristics (23–25). Despite the widespread attention to the role of BDNF in SZ, its clinical utility remains limited due to inconsistent findings.

In exploring reliable biomarkers for SZ, the neuroendocrine system has attracted considerable attention due to its regulatory role in physiological processes. Notably, norepinephrine (NE) and melatonin (MLT) have been implicated in neuroendocrine disorders including SZ. NE, synthesized in the locus coeruleus, plays a crucial role in the body’s stress response by activating the hypothalamic-pituitary-adrenal axis (26, 27). It influences attention, arousal, and cognitive functions, which are often impaired in SZ (28, 29). Altered NE levels have been associated with mood disorders and cognitive deficits, indicating its involvement in SZ pathogenesis (30). Similarly, MLT, produced by the pineal gland, regulates circadian rhythms and possesses neuroprotective properties (31). Disruptions in MLT secretion have been linked to sleep disturbances and mood disorders, common in patients with SZ (32, 33). Additionally, MLT modulates the neuroendocrine system and immune responses, underscoring its role in maintaining physiological equilibrium (34). These insights suggest that NE and MLT may contribute to SZ pathophysiology, presenting them as potential biomarkers for the disorder. Our study aimed to identify more biomarkers by comparing metabolites in healthy individuals and patients with SZ using high-throughput analyses, revealing significant differences in hundreds of small molecules.

## Methods

### Participants

This study received approval from the Ethics Committee of Pudong New Area Mental Health Center, an affiliate of Tongji University, demonstrating adherence to ethical standards. A total of 38 female patients diagnosed with SZ, in accordance with Diagnostic and Statistical Manual of Mental Disorders, Fourth Edition (DSM-IV) criteria, were recruited from Pudong New District Mental Health Center. To eliminate gender-related influences, all recruited patients were female. All the blood samples were collected at 8:00 a.m. The exclusion criteria for patients included: 1) a documented history of other mental disorders, 2) the presence of neurological diseases or other significant medical conditions, such as tumors, 3) acute exacerbation of psychotic symptoms, and 4) a history of drug abuse within the preceding 30 days.

Furthermore, 38 healthy female individuals from the community were enlisted to serve as the control group. The exclusion criteria for the control group were as follows: 1) a history of psychosis or neurological disorders, 2) a family history of mental disorders, 3) the presence of other severe medical conditions, and 4) a lifetime history of substance abuse. All participants provided written informed consent, ensuring their voluntary participation in the study.

### Chemicals

All chemicals and solvents employed in this study were of analytical or high-performance liquid chromatography (HPLC) grade. Acetonitrile and methanol were procured from Thermo Fisher Scientific (Waltham, MA, USA). Pyridine, n-hexane, methoxylamine hydrochloride (97% purity), and bis(trimethylsilyl) trifluoroacetamide (BSTFA) containing 1% trimethylchlorosilane (TMCS) were obtained from CNW Technologies GmbH (Düsseldorf, Germany). L-2-chlorophenylalanine was sourced from Shanghai Hengchuang Biotechnology Co., Ltd. (Shanghai, China).

### GC-MS sample preparation and analysis

For GC-MS analysis, serum samples stored at −80°C were thawed at room temperature, and 150μl of each sample was transferred to a 1.5mL centrifuge tube. A volume of 450μl of pre-cooled protein precipitation solution (methanol: acetonitrile = 2:1, v/v) was added, followed by vortex mixing for 1min. Samples were then subjected to ultrasonic extraction in an ice-water bath for 10min, held at −40°C for 30min, and centrifuged at 12,000rpm for 10min at 4°C. A total of 150μl of the supernatant was collected and dried using a vacuum centrifugal concentrator. To the dried residue, 80μl of methoxylamine hydrochloride in pyridine (15mg/mL) was added for oximation at 37°C for 60min. Subsequently, 50μl of BSTFA (containing 1% TMCS), 20μl of n-hexane, and 10μl of an internal standard mixture were added. The derivatization was carried out at 70°C for 60min, followed by equilibration at room temperature for 30min prior to GC-MS metabolomics analysis.

GC-MS analysis was performed on an Agilent 7890B gas chromatograph coupled to a 5977A mass spectrometer. Metabolites were separated using a DB-5MS capillary column (30m × 0.25mm × 0.25μm; Agilent J&W Scientific, USA). High-purity helium (≥99.999%) was used as the carrier gas at a constant flow rate of 1.0mL/min. The injection temperature was set to 260°C, with a 1μl injection volume in splitless mode and a solvent delay of 6.2min. The oven temperature program was as follows: initial temperature of 60°C for 0.5min, ramped to 125°C at 8°C/min, to 210°C at 8°C/min, to 270°C at 15°C/min, and finally to 305°C at 20°C/min, held for 5min. Mass spectrometry was conducted in electron ionization (EI) mode with an ion source temperature of 230°C, quadrupole temperature of 150°C, electron energy of 70eV, and a mass scan range of m/z 50–500 in full scan (SCAN) mode.

### LC-MS sample preparation and analysis

For LC-MS analysis, samples stored at −80°C were thawed at room temperature, and 150μl was transferred to a 1.5mL Eppendorf tube. Then, 450μl of pre-cooled protein precipitation solution (methanol: acetonitrile = 2:1, v/v) was added, followed by vortexing for 1min. The mixture was subjected to ultrasonic extraction in an ice-water bath for 10min, then allowed to stand at −40°C for 2h. After centrifugation at 12,000rpm for 10min at 4°C, 150μl of the supernatant was withdrawn using a syringe and filtered through a 0.22μm organic-phase syringe filter. The filtrate was transferred to LC vials and stored at −80°C until analysis. All extraction reagents were pre-cooled to −20°C before use. Quality control (QC) samples were prepared by pooling equal aliquots from all sample extracts.

LC-MS analysis was performed using an ACQUITY UPLC I-Class Plus system coupled with a Q Exactive high-resolution mass spectrometer. Chromatographic separation was achieved on an ACQUITY UPLC HSS T3 column (100mm × 2.1mm, 1.8μm) maintained at 45°C. The mobile phases consisted of water with 0.1% formic acid (A) and acetonitrile (B), delivered at a flow rate of 0.35mL/min. The injection volume was 5μl.

### Data preprocessing and statistical analysis

The obtained GC-MS and LC-MS raw data were transferred via software Analysis Base File Converter for quick retrieval of data. Then, data were imported into software MS-DIAL, which performs peak detection, peak identification, MS2Dec deconvolution, characterization, peak alignment, wave filtering, and missing value interpolation. Metabolite characterization of GC-MS is based on NIST Chemistry WebBook. Metabolite characterization of LC-MS is based on The Human Metabolome Database(HMDB)、Lipidmaps(v2.3)and METLIN database (35–37). A data matrix was derived. The three-dimensional matrix includes: sample information, the name of the peak of each substance, retention time, retention index, mass-to-charge ratio, and signal intensity. In each sample, all peak signal intensities were segmented and normalized according to the internal standards with RSD greater than 0.3 after screening. After the data was normalized, redundancy removal and peak merging were conducted to obtain the data matrix. The matrix was imported in R to carry out principle component analysis (PCA) to observe the overall distribution among the samples and the stability of the whole analysis process.

Orthogonal Partial Least-Squares-Discriminant Analysis (OPLS-DA) and Partial Least-Squares-Discriminant Analysis (PLS-DA) were utilized to distinguish the metabolites that differ between groups. To prevent overfitting, 7-fold cross-validation and 200 Response Permutation Testing (RPT) were used to evaluate the quality of the model. Variable Importance of Projection (VIP) values obtained from the OPLS-DA model were used to rank the overall contribution of each variable to group discrimination. A two-tailed Student’s T-test was further used to verify whether the metabolites of difference between groups were significant. Differential metabolites were selected with VIP values greater than 1.0 and p-values less than 0.05.

### Enzyme linked immunosorbent assay

ELISA assays for BDNF were performed on serum samples from 25 healthy controls and 24 patients with SZ. For the detection of norepinephrine (NE) and melatonin (MLT), serum samples from 38 healthy controls and 38 patients with SZ were analyzed using ELISA.

The ELISA kits of BDNF, NE and MLT were brought form abcam (ab212166, ab287789, ab283259). The specific experimental procedures were conducted in accordance with the instructions provided. In summary, all reagents, samples, and standard substances were prepared as per the manufacturer’s guidelines. Subsequently, the plate was washed with Wash Solution. Then, standard substances or samples were added into the respective wells, followed immediately by the addition of the Biotin-detection antibody working solution. Then, the plate was sealed and incubated at 37°C for 45 minutes. The solution was discarded, and the plate was washed with the Wash Solution. Following the final wash, any remaining Wash Solution was removed by aspiration using absorbent filter papers. Subsequently, SABC working solution was added to each well, and the plate was covered and incubated at 37°C for 30 minutes. After discarding the solution, the plate was washed with Wash Solution. Next, TMB substrate was added to each well, and the plate was covered and incubated at 37°C in darkness. Finally, Stop Solution was added, and the results were read at 450 nm within a 20-minute timeframe.

### Statistical analyses

The differences in metabolite levels between the two groups were analyzed using GraphPad Prism 9 software. Subsequent comparisons between the groups were performed using two-tailed Student’s t-tests. All experimental data are presented as mean ± standard error of the mean (SEM) and are representative of three independent experiments. A p-value of less than 0.05 was considered statistically significant. Asterisks denote significance levels as follows: *p < 0.05, **p < 0.01, and ***p < 0.001.

## Results

### Evaluating the limitations of BDNF and exploring feasible biomarkers in SZ

**Figure 1:**
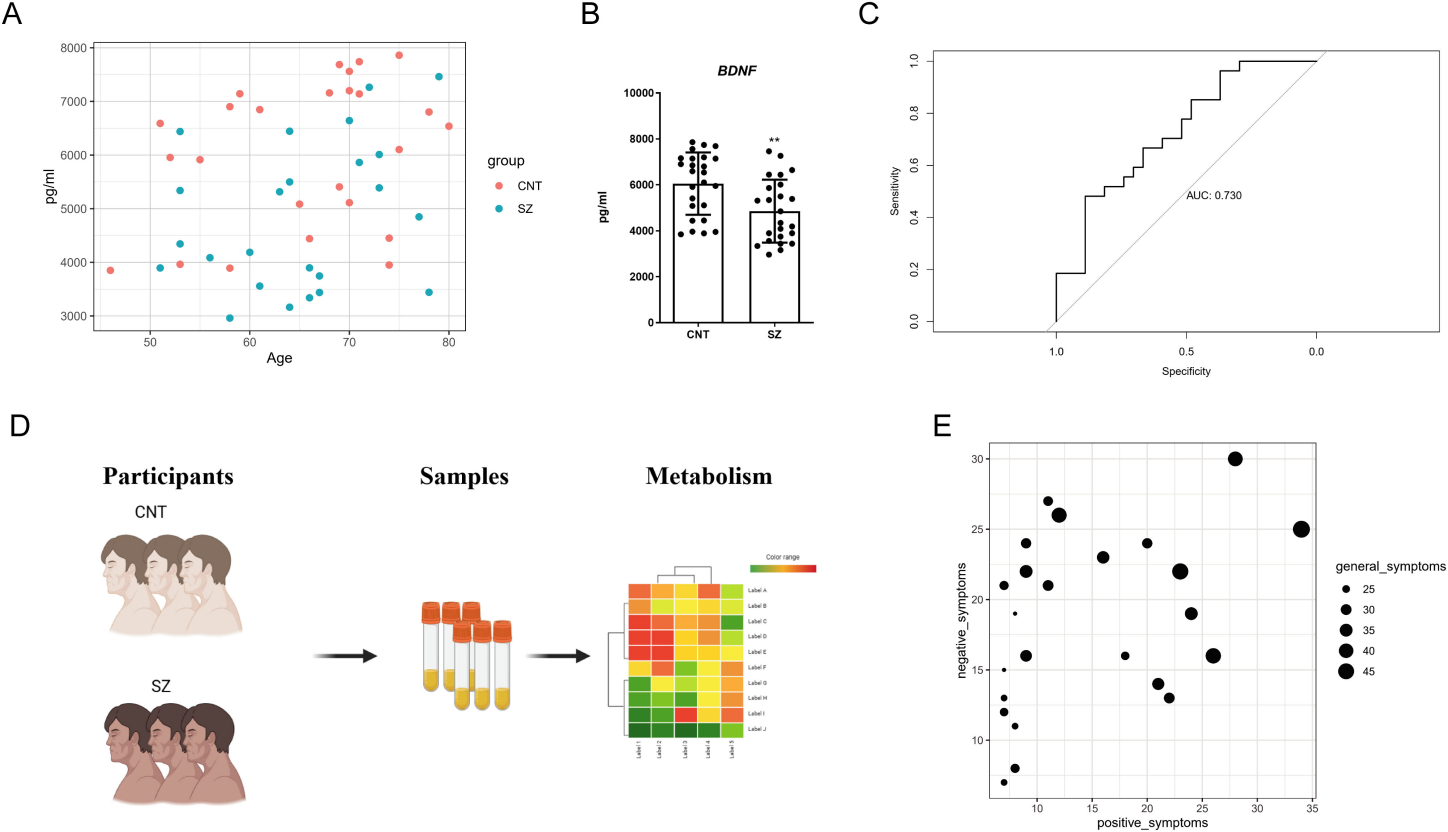
Evaluating the limitations of BDNF and Schematic workflow of the study **(A)** Scatter plot depicting BNDF levels in the plasma of schizophrenia (SZ) patients versus Healthy control (CNT) groups. It displays the relationship between Age (x-axis) and BNDF concentration (pg/ml) (y-axis). Each point represents a subject, with red dots for the CNT group and blue dots for the SZ group. **(B)** Bar chart summarizing the statistical analysis of BNDF protein levels in the plasma between patients with SZ and Healthy control (CNT). The error bars represent the standard error of the mean (SEM). Asterisks (**) indicate p < 0.01. **(C)** Receiver Operating Characteristic (ROC) curve illustrating the diagnostic potential of BNDF for patients with SZ. The x-axis represents 1 - Specificity (false positive rate), while the y-axis represents Sensitivity (true positive rate). The Area Under the Curve (AUC) is 0.730, suggesting that the model has only modest discriminative ability. **(D)** Schematic diagram outlining the study's research strategy. Participants were divided into two groups: healthy controls (CNT) and individuals with SZ. Biological samples (serum) were collected from both groups, which were then subjected to metabolic profiling. **(E)** Negative, positive, and general symptoms of participants with SZ.

BDNF is currently one of the most extensively studied biomarkers for schizophrenia. To evaluate its efficacy, we collected serum samples from 25 healthy females and 24 female schizophrenia patients. Upon analysis, we found that the distribution of BDNF levels in both groups were broad (**Figure 1A**). Statistical analysis showed a certain level of significance, but the median difference between the two groups was not large (**Figure 1B**). Further evaluation using the curve analysis indicated an AUC value of only 0.73, which is less than 0.8, suggesting that BDNF is not an ideal discriminative marker (**Figure 1C**). These results imply that although BDNF levels are altered in schizophrenia patients, it is not a reliable distinguishing marker. For the identification of feasible biomarkers, we collected blood samples from individuals with SZ. Using both Gas Chromatography-Mass Spectrometry (GC-MS) and Liquid Chromatography-Mass Spectrometry (LC-MS) techniques, we profiled samples from 24 female patients with SZ and 25 healthy female control subjects (**Figure 1D**, **Supplementary Figure S1**). We then evaluated the patients for negative, positive, and general symptoms (**Figure 1E**). As **Figure 1E** illustrates, patients experiencing severe negative and positive symptoms also faced more severe general symptoms, and vice versa.

### Metabolites exhibit significant differences in patients with SZ and healthy individuals

**Figure 2:**
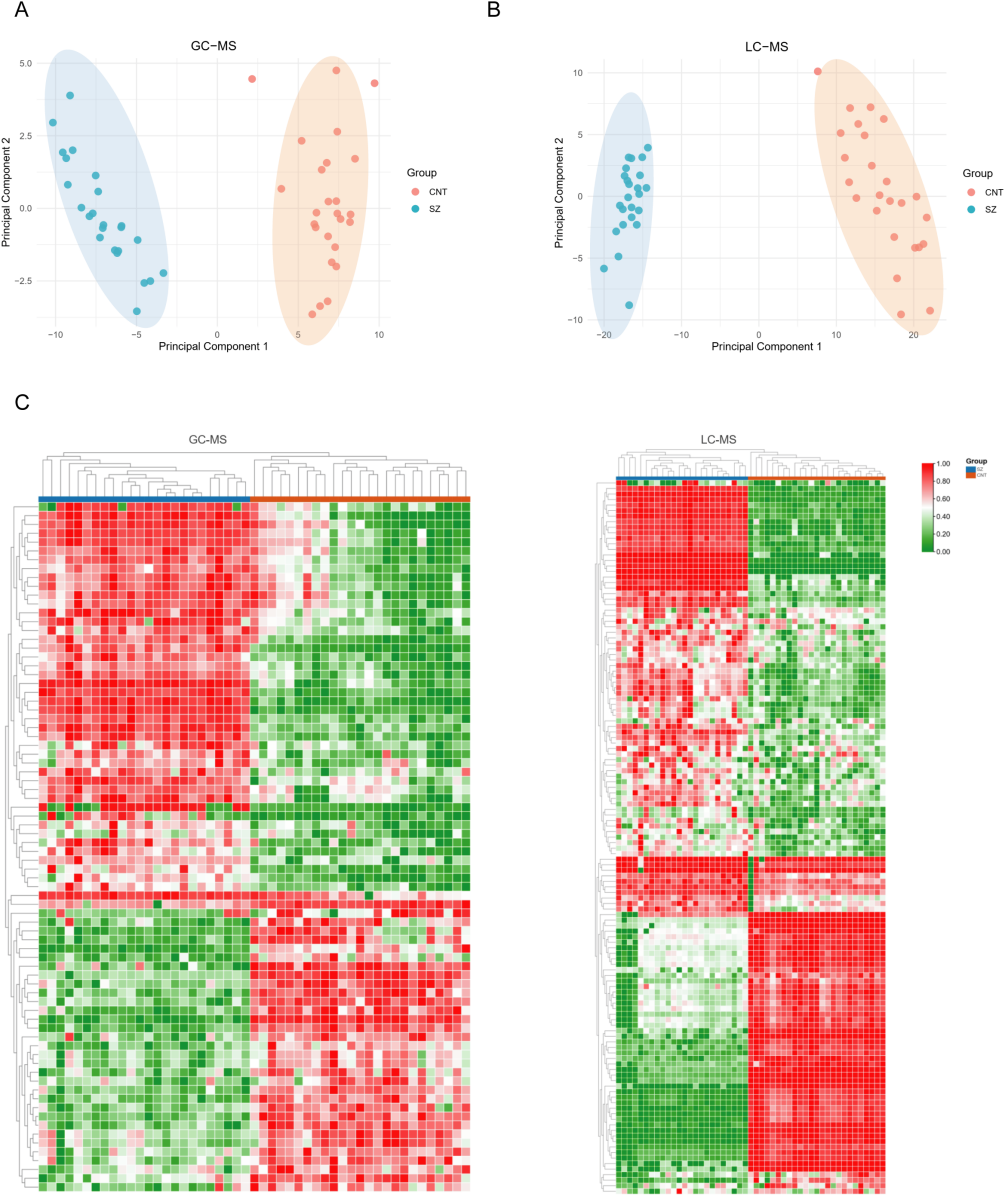
Metabolites exhibit significant differences in patients with SZ and healthy individuals **(A, B)** Principal Component Analysis (PCA) plots illustrate the metabolic profiling results obtained from GC-MS **(A)** and LC-MS **(B)** platforms. In both PCA plots, the CNT and SZ groups form distinct clusters with minimal overlap, suggesting clear metabolic differences between the two groups. **(C)** Heatmaps the metabolic profiles obtained using GC-MS (left) and LC-MS (right) platforms. In both panels, each column represents an individual sample, and each row represents a metabolite. Samples are grouped into CNT (blue) and SZ (red).

We applied PCA to assess the overall characteristics of the acquired data (**Figures 2A, B**), revealing significant disparities – notably clear distinctions between patients with SZ and healthy individuals. To highlight these metabolic differences, we utilized a heatmap, which demonstrates distinctions between the SZ and control groups based on the top fifty significantly altered metabolites (**Figure 2C**). Additionally, these metabolites showed considerable intra-group variation, indicating their potential as specific SZ biomarkers.

### Significantly differentiated metabolites in SZ

**Figure 3:**
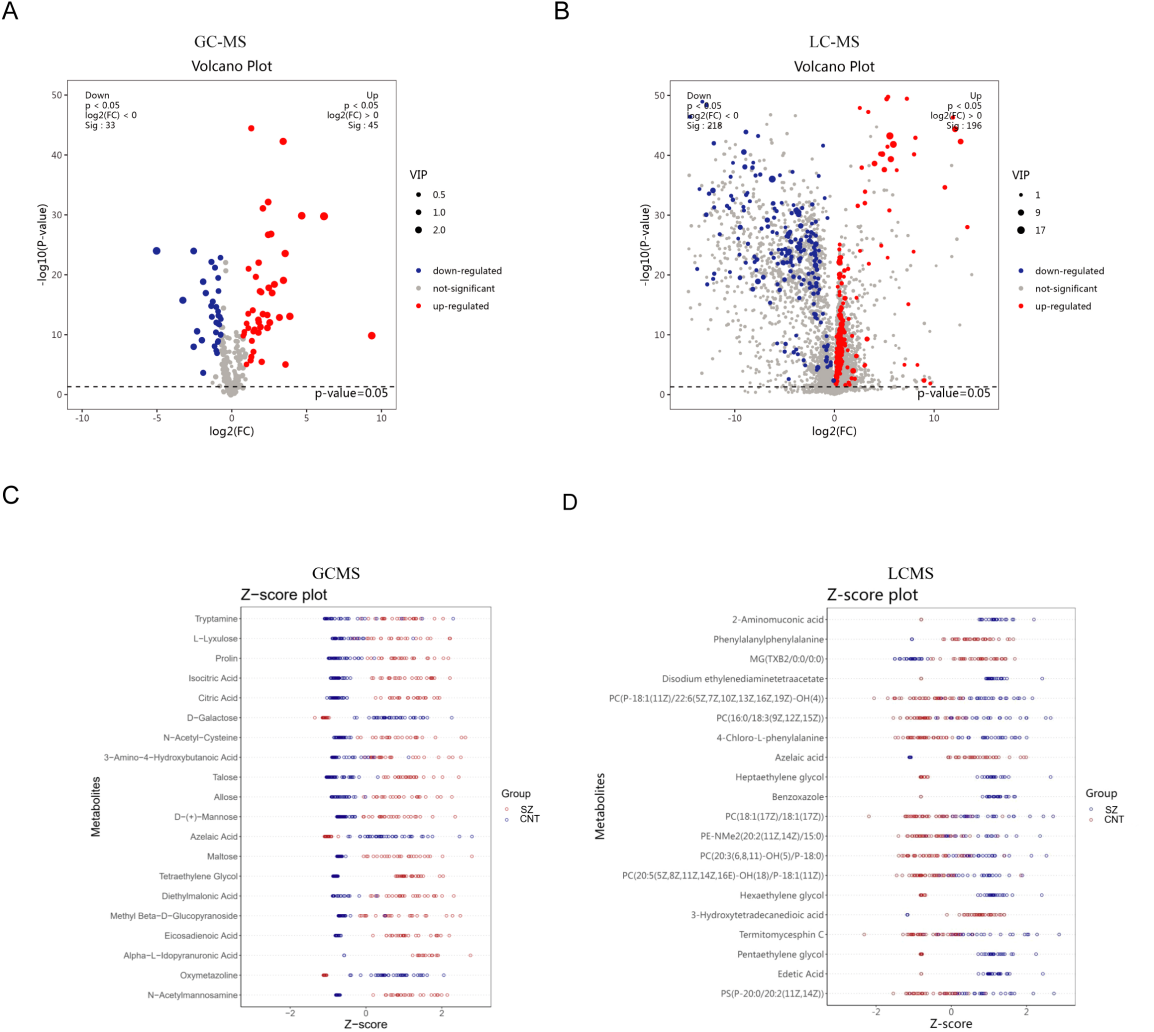
Significantly differentiated metabolites in patients with SZ and healthy individuals **(A, B)** Volcano plots illustrate differential metabolites between the CNT group and the SZ group from GC-MS **(A)** and LC-MS **(B)** platforms. **(C, D)** The top 20 significantly altered metabolites within the groups from GC-MS **(C)** and LC-MS **(D)** platforms.

Using a volcano plot, we identified metabolites with significant changes (**Figures 3A, B**). The GC-MS results indicate 79 major differential metabolites, while LC-MS revealed 419. These results confirm the presence of many significant differential metabolites in patients with SZ. We further highlighted the top 20 significantly altered metabolites to illustrate their variance within the groups (**Figures 3C, D**). GC-MS analysis revealed a significant elevation of serum tryptamine in schizophrenia patients. As an amine derivative in the tryptophan metabolic pathway and a precursor to neuroendocrine hormones such as serotonin and melatonin, tryptamine may modulate mood and cognitive functions. In contrast, azelaic acid—a metabolite derived from the oxidation of unsaturated fatty acids and associated with oxidative stress and inflammatory processes—was significantly reduced. LC-MS analysis further showed a significant increase in 2-aminomuconic acid, an intermediate of the kynurenine pathway, dysregulation of which may contribute to neurotoxicity and altered glutamate receptor function. Notably, the reduction of azelaic acid in LC-MS results corroborates the GC-MS findings.

### Functional clustering analysis of differential metabolites

**Figure 4:**
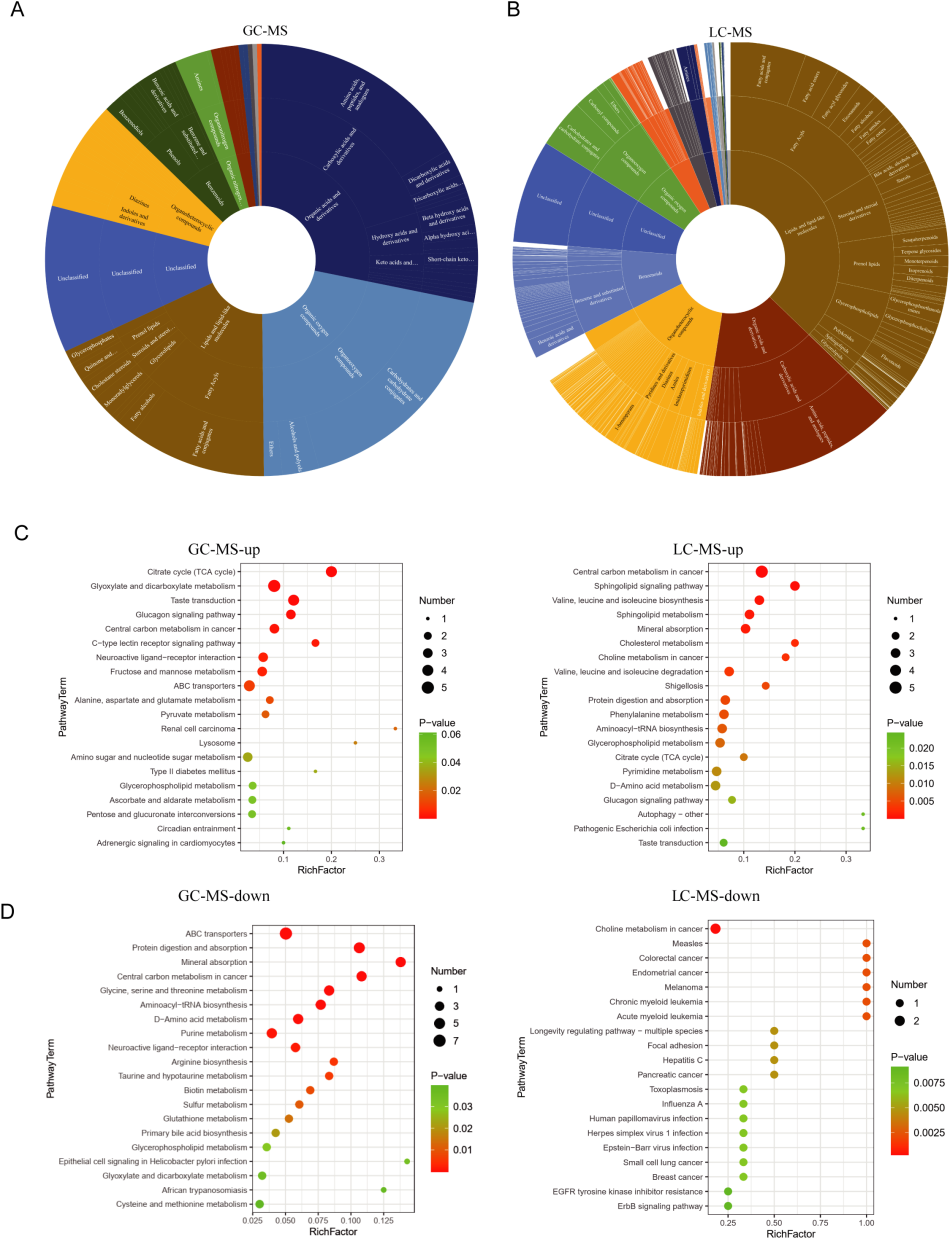
Functional clustering analysis of differential metabolites **(A, B)** the biological functions associated with relevant differential small metabolites. In the circular diagrams, the innermost circle represents the major categories, followed by subcategories in the middle, and subclasses on the outermost ring. **(C)** functional clustering analysis of upregulated metabolites is presented. On the left side, the biological functions associated with the upregulated metabolites identified in GC-MS are displayed. On the right side, the corresponding metabolite count and P-values for each function are further presented. Panel B illustrates the results obtained from LC-MS. **(D)** functional clustering analysis of downregulated metabolites is presented. On the left side, the metabolites identified in GC-MS. On the right side, the corresponding metabolite obtained from LC-MS.

We analyzed the biological functions and associated pathways of these differential metabolites. The prevalent categories include organic acids, lipids, and oxidative organic compounds in both GC-MS and LC-MS results (**Figures 4A, B**). Among the pathways identified as altered in the GC-MS analysis, the neuroactive ligand–receptor interaction pathway encompasses a broad spectrum of neurotransmitters and their receptors, including dopamine, glutamate, and GABA. This pathway is considered a central pathogenic mechanism in schizophrenia. The alanine, aspartate, and glutamate metabolism pathways are closely linked to glutamate metabolism, which is critical given glutamate’s role as the primary excitatory neurotransmitter in the central nervous system. Likewise, the choline metabolism in cancer pathway is related to choline metabolism, and disruptions in the cholinergic system have been associated with attentional deficits and cognitive impairments frequently observed in individuals with schizophrenia (**Figure 4C**). Interestingly, the decreased metabolites in GC-MS related to various diseases, such as breast cancer and leukemia (**Figure 4D**), which might be due to the limited metabolite-pathway association. We noted the involvement of glutathione metabolism, a critical pathway in cellular antioxidant defense (**Figure 4D**). Previous studies have demonstrated elevated markers of oxidative stress and reduced glutathione levels in the brains of individuals with schizophrenia. These results suggest that changes in small metabolites primarily involve metabolic and neural-related functions.

### MLT and NE is promising neuroendocrine markers for diagnosing SZ

**Figure 5:**
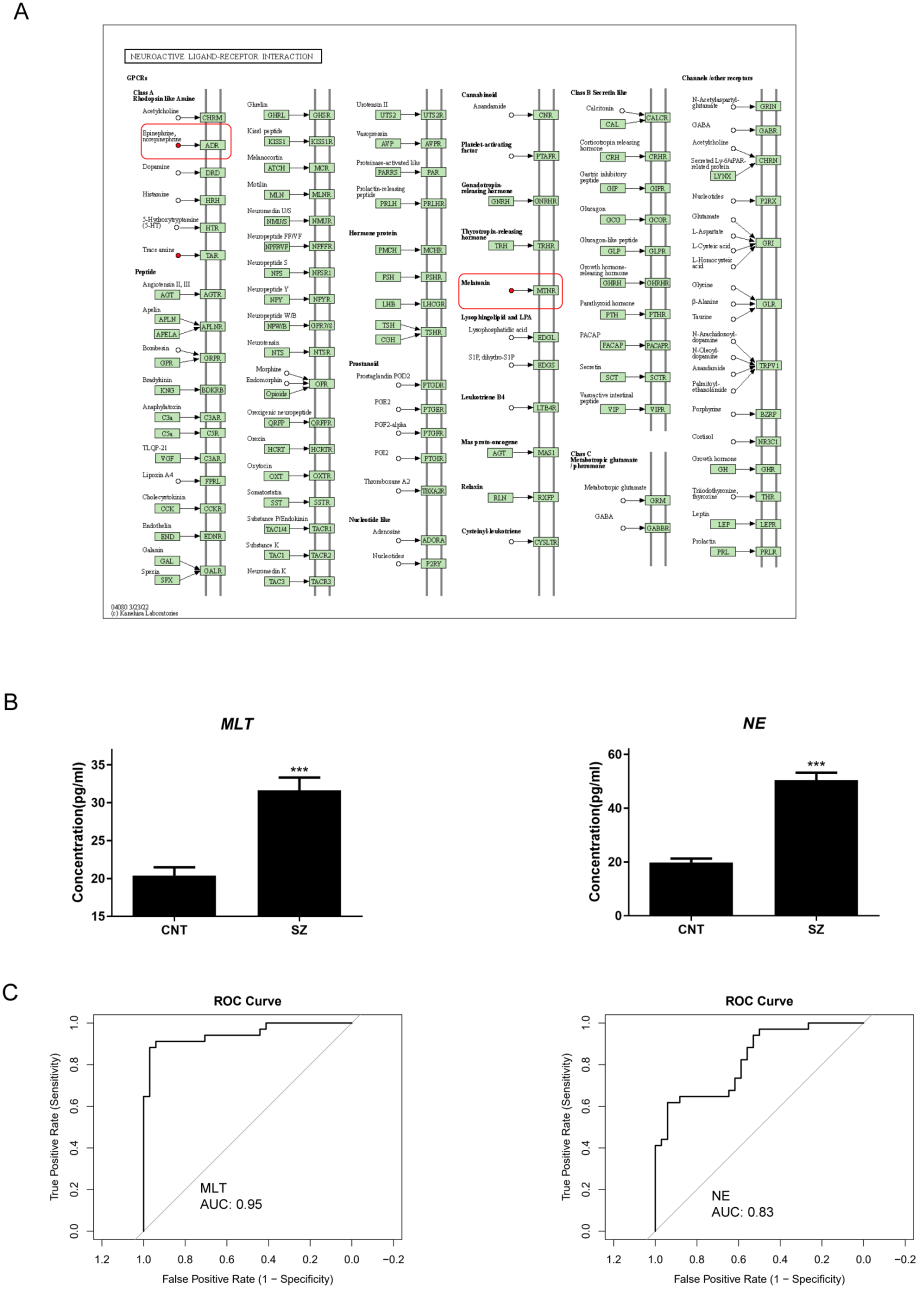
Norepinephrine and Melatonin are prominent neuroendocrine marker in SZ **(A)** right: the KEGG pathway mapper displays differential metabolic pathways and color-codes differential metabolites based on their upregulation and downregulation information. Small circles in the metabolic pathway map represent metabolites. Metabolites highlighted in red in the pathway map indicate upregulated metabolites detected in the experiment. **(B)** The variations in concentration of Norepinephrine (NE) and Melatonin (MLT) between patients with Schizophrenia (SZ) and Healthy control (CNT). The error bars represent the standard error of the mean (SEM). Asterisks (***) indicate p < 0.001. **(C)** Receiver Operating Characteristic (ROC) curve illustrating the diagnostic potential of NE (left) and MLT (right) for patients with SZ. The x-axis represents 1 - Specificity (false positive rate), while the y-axis represents Sensitivity (true positive rate). The Area Under the Curve (AUC) is over 0.8, suggesting that the model has well discriminative ability.

Analysis revealed remarkable elevations in the neuroendocrine metabolites, NE and MLT (**Figure 5A**). ELISA results confirmed these findings, with patients with SZ displaying notably higher concentrations of NE and MLT compared to healthy subjects (**Figure 5B**). Analyzing Receiver Operating Characteristic (ROC) curves revealed that the value of Area Under Curve (AUC) were both higher than 0.8 (**Figure 5C**). Notably, their superior AUC values relative to BDNF underscore their potential to serve as more dependable biomarkers for SZ. Thus, our findings affirm the potential of these neuroendocrine markers for SZ diagnosis.

## Discussion

Despite numerous years of intense research, the pathophysiology of SZ is yet uncharted (38, 39). In this study, we evaluated the potential of neuroendocrine and metabolic markers in SZ, emphasizing BDNF’s limitations and highlighting the potential of NE and MLT. BDNF has long been considered one of the most extensively studied biomarkers for SZ, yet previous reports of inconsistent clinical utility (23, 25). Our findings demonstrated that its diagnostic performance in our cohort was suboptimal (AUC <0.8). This underscores the need for alternative biomarkers that can offer both better specificity and sensitivity. Among the neuroendocrine markers we investigated, NE and MLT emerged as strong candidates for SZ diagnosis. Firstly, compared to BDNF, the serum levels of NE and MLT in patients with SZ are significantly higher than those in the controls; secondly, both were significantly elevated in patients with SZ compared to controls, and their AUC values exceeded 0.8—substantially outperforming BDNF in our cohort. By demonstrating stronger diagnostic potential, NE and MLT appear to address the current gap in reliable biomarkers for SZ, offering a more consistent measure of the pathophysiological changes underlying this disorder.

MLT is an indole hormone secreted by the pineal gland (40). Its secretion is regulated by the light-dark cycle, with a marked increase during the night and suppression by light during the day, thereby modulating the sleep–wake cycle. MLT modulates several signaling pathways, including the AC/cAMP pathway, PLC/Ca²^+^ signaling, and PI3K/AKT pathways, primarily through its G protein-coupled receptors, MT1 and MT2 (41, 42). In neurons, MLT activates its receptor and decreases adenylate cyclase activity, leading to reduced intracellular cAMP levels. This reduction modulates ion channel activity and neurotransmitter release, thereby depressing neuronal excitability and synaptic plasticity (43). ​Given MLT’s pivotal role in regulating circadian rhythms, neurotransmission, and neuroprotection, its dysfunction is closely associated with the development of various psychiatric disorders (44). For example, abnormal MLT secretion can lead to sleep disturbances and impaired mood regulation, increasing the risk of anxiety and depression. Similarly, the total score of the Brief Psychiatric Rating Scale of patients with SZ is positively correlated with MLT concentration (45). We also found that MLT levels were significantly elevated in patients with SZ. Previous studies have demonstrated that serum MLT concentrations gradually decline with age. Consistently, we observed a similar age-related trend (**Supplementary Figure S2**); however, this decline did not affect the elevated MLT levels observed in patients with SZ (**Figure 5B**). In addition to its diagnostic potential, this marker may also have significant therapeutic applications. Sleep dysfunction is a common problem in people with SZ, MLT therapy has some positive outcomes for sleep, metabolic profile and tardive dyskinesia in patients with SZ (46).

NE, a hormone and an important neurotransmitter, is primarily synthesized and released in the brain by the locus coeruleus in the brainstem, as well as by peripheral sympathetic neurons (47). It exerts its effects through adrenergic receptors, activating distinct downstream signaling pathways, such as the PLC/IP3/Ca²^+^, AC/cAMP/PKA, and MAPK/ERK cascades (48). These pathways regulate cellular excitability, gene expression, and synaptic transmission, playing critical roles in modulating emotion, stress responses, and cognitive functions. Dysregulation of these signaling mechanisms is considered a key molecular basis underlying various psychiatric disorders, including anxiety, depression, and Post-Traumatic Stress Disorder (PTSD) (49, 50). The progression of SZ is closely associated with NE. For example, NE dysregulation has been implicated in the pathophysiology of SZ, with elevated NE levels in patients (51). Notably, NE receptors play a crucial role in the dysregulation of cognitive, arousal, and emotional systems associated with SZ (52). Additionally, highly selective adrenergic agents may be useful for enhancing prefrontal cortex function in patients with SZ (53). These research, together with our results, emphasize the promising value of NE dysregulation in SZ.

Metabolic dysregulation has emerged as a critical component in the pathophysiology of SZ (54, 55). Aberrant glutamatergic signaling is recognized as a key driver of disease etiology, with multiple studies reporting significant alterations in glutamate and related amino acids in both blood and cerebrospinal fluid (56, 57). In our study, we observed a marked elevation in Alanine, aspartate and glutamate metabolism (**Figure 4**), suggesting that an imbalance between excitatory and inhibitory neurotransmission. Similarly, markers of oxidative stress are elevated in SZ since antioxidant capacity is reduced (58). Consistently, we also observed diminished antioxidant capacity, a notable reduction in glutathione metabolism (**Figure 4**). This reduction may compromise neuronal membrane integrity, leading to the limited regenerative potential of neurons. Collectively, these findings underscore the pivotal role of metabolic perturbations—particularly within glutamatergic and oxidative stress pathways—in the complex pathogenesis of SZ.

BDNF, MLT, and NE are closely interconnected in regulating neural functions, particularly in cognition, sleep, and emotional processes. NE enhances BDNF expression by activating β-adrenergic receptors, which induces BDNF production in hippocampal neurons and stimulates pathways, including PI-3K/Akt and MAPK signaling (59). Additionally, NE plays a critical role in the regulation of MLT secretion by stimulating β-adrenergic receptors, thereby modulating the circadian rhythm (60). Besides, MLT influences BDNF levels by upregulating SIRT1 expression, contributing to neuroprotection (61). These interactions underscore the synergistic role of BDNF, MLT, and NE in maintaining neurological health. However, disruptions in the balance of BDNF, MLT, and NE—such as reduced BDNF and elevated MLT and NE—may contribute to SZ-related changes, including impaired synaptic plasticity, dysregulated sleep-wake cycles, and emotional instability. These imbalances could be involved in SZ pathogenesis, warranting further investigation.

This study, while informative, has limitations. It consists of a modest sample size, and with SZ’s substantive heterogeneity, distinct subtypes, and clinical stages, diverse metabolic patterns abound. Therefore, replication with a larger cohort can elevate the statistical robustness and generalizability of these findings. Additionally, the cross-sectional study design precludes inferences about causal relationships between the metabolic aberrations and SZ. Further, the cohort was exclusively female; thus, the application of the findings to male patients with SZ is unknown. Future research should circumscribe both genders to examine potential gender-mediated metabolic distinctions in SZ. To validate the clinical findings, it is necessary to perform cellular and animal experiments. Additionally, to validate these clinical findings, it is essential to perform cellular and animal experiments.

In summary, our study provides innovative insights into the metabolic and neuroendocrine patterns associated with SZ, specifically in female patients. Though limitations exist, such as sample size and gender bias, these findings lay the groundwork for additional exploration into the pathophysiology of SZ and potential diagnostic and therapeutic biomarkers.

## Data Availability

The data that support the findings of this study are available on reasonable request from the corresponding author.
