# Supplementary figures and images for: Neuroendocrine characterization into schizophrenia: norepinephrine and melatonin as promising biomarkers
Source: Front Endocrinol (Lausanne). 2025 May 1;16:1551172. doi: 10.3389/fendo.2025.1551172 (PMC12078152; doi:10.3389/fendo.2025.1551172)

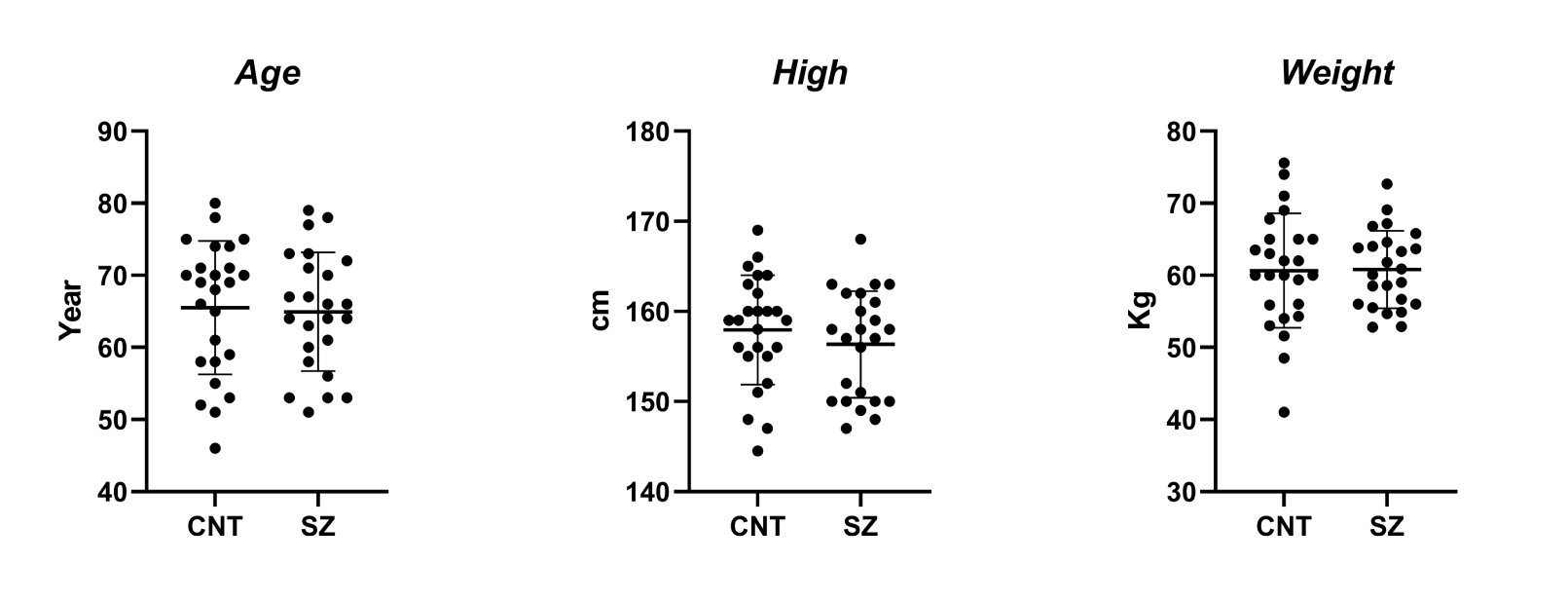

Supplement: Supplementary file 1 [file Image1.tif]

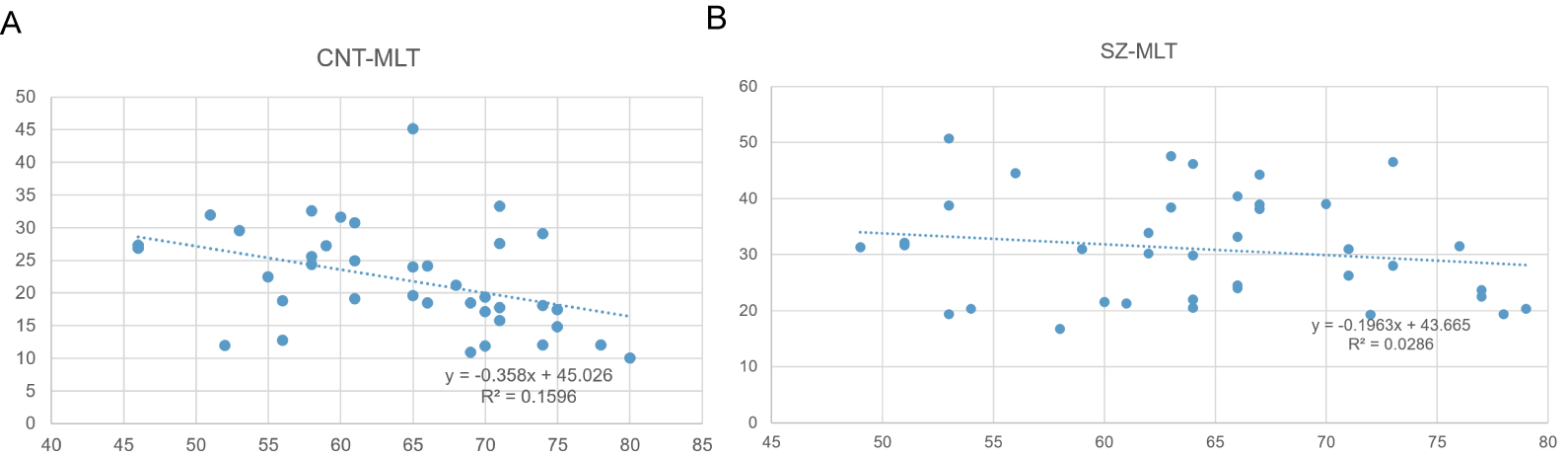

Supplement: Supplementary file 2 [file Image2.tif]
